# End systolic volume and scar burden are incremental and independent predictors of survival in patients with severe ischemic cardiomyopathy

**DOI:** 10.1186/1532-429X-14-S1-O16

**Published:** 2012-02-01

**Authors:** Deborah Kwon, Rory Hachamovitch, Zoran B Popovic, Scott D Flamm, Thomas Marwick

**Affiliations:** 1 Cleveland Clinic, Cleveland, OH, USA

## Background

Scar burden has been shown to be an independent predictor of mortality in patients with severe ischemic cardiomyopathy (ICM). However, it is unclear how both scar burden and end systolic volume (ESV) impact outcomes in patients with severe ischemic (ICM).

### Purpose

In patients with severe ICM, we sought to assess the association of ESV and scar burden with outcomes in severe ICM.

## Methods

450 patients with > 70% stenosis in ≥1 epicardial coronary artery (75% men, median age 63 years, median LV ejection fraction (EF) 22%, median ESVi 106ml, median scar % of 29%) underwent delayed hyperenhancement-MRI (Siemens 1.5-T scanner, Erlangen, Germany) between 2003-2007. CMR evaluation included long and short axis assessment of LV function on balanced steady state free precession images along with assessment of myocardial scar (on phase-sensitive inversion recovery DHE-CMR sequence ~ 10-20 minutes after injection of 0.2 mmol/kg of Gadolinium dimenglumine). Scar was identified as regions of interest > 2 SD above normal myocardium. LV scar was was recorded as a percentage of the total myocardium and transmural extent (0 = none, 1 = 1-25%, 2 = 26-50%, 3 = 51-75%, and 4 = > 75%). Total scar score was determined from the summed scar score of 17 segments per patient divided by 17. Cox proportional hazards survival modeling, using a primary end-point of all-cause mortality, was used to risk-adjust comparisons.

## Results

**Figure 1:**
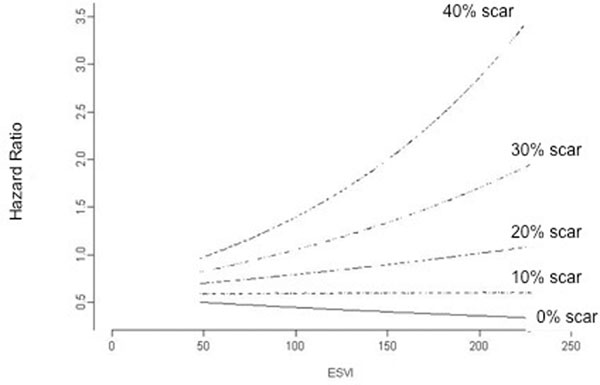


Over a follow-up of up to 9 years [mean 5.75years], 186 deaths occurred. Survival analysis revealed that after adjusting for subsequent CABG, sex, age, and mitral valve procedures, indexed ESV (ESVi) (χ2 19.25, p = 0.0017) and scar % (χ2 25.97, p = <0.001) were independent and additive predictors of all cause mortality. Higher ESVI, in the setting of higher scar %, resulted in significantly worse mortality than in patients with higher scar % and lower ESVi. On the other hand, higher ESVi did not significantly impact survival in patients with minimal or no myocardial scar. (See Figure 1).

## Conclusions

ESVi and scar burden provide independent and incremental prognostic value in patients with severe ICM. Prognosis should not be considered by scar burden in isolation.

## Funding

None.

